# Laboratory testing of a shuttle car canopy air curtain for respirable coal mine dust control

**DOI:** 10.1007/s40789-018-0225-2

**Published:** 2018-09-17

**Authors:** W. R. Reed, Y. Zheng, M. Yekich, G. Ross, A. Salem

**Affiliations:** 1 Pittsburgh Mining Research Division, Centers for Disease Control and Prevention, National Institute for Occupational Safety and Health, 626 Cochrans Mill Rd., Pittsburgh, PA 15236 USA; 2 J.H. Fletcher, Inc., Huntington, WV USA; 3 Marshall University, Huntington, WV USA

**Keywords:** Shuttle car, Canopy air curtain, Dust, Airflow, Coal mining

## Abstract

Canopy air curtain (CAC) technology has been developed by the National Institute for Occupational Safety and Health (NIOSH) for use on continuous miners and subsequently roof bolting machines in underground coal mines to protect operators of these machines from overexposure to respirable coal mine dust. The next logical progression is to develop a CAC for shuttle cars to protect operators from the same overexposures. NIOSH awarded a contract to Marshall University and J.H. Fletcher to develop the shuttle car CAC. NIOSH conducted laboratory testing to determine the dust control efficiency of the shuttle car CAC. Testing was conducted on two different cab configurations: a center drive similar to that on a Joy 10SC32AA cab model and an end drive similar to that on a Joy 10SC32AB cab model. Three different ventilation velocities were tested—0.61, 2.0, 4.3 m/s (120, 400, and 850 fpm). The lowest, 0.61 m/s (120 fpm), represented the ventilation velocity encountered during loading by the continuous miner, while the 4.3 m/s (850 fpm) velocity represented ventilation velocity airflow over the shuttle car while tramming against ventilation airflow. Test results showed an average of the dust control efficiencies ranging from 74 to 83% for 0.61 m/s (120 fpm), 39%–43% for 2.0 m/s (400 fpm), and 6%–16% for 4.3 m/s (850 fpm). Incorporating an airflow spoiler to the shuttle car CAC design and placing the CAC so that it is located 22.86 cm (9 in.) forward of the operator improved the dust control efficiency to 51%–55% for 4.3 m/s (850 fpm) with minimal impact on dust control efficiencies for lower ventilation velocities. These laboratory tests demonstrate that the newly developed shuttle car CAC has the potential to successfully protect shuttle car operators from coal mine respirable dust overexposures.

## Introduction

The development of the canopy air curtain (CAC) dates back to the 1970s starting with the initial development of the CAC by the Donaldson Company, Inc. under contract from the U.S. Bureau of Mines (Krisko 1975). This CAC was originally developed for continuous miner operators when continuous mining machines had cabs. The need for a CAC on the continuous miner was eliminated when the cab was removed from the machine design. However, CAC development progressed to include CAC designs for a roof bolting machine to protect roof bolters from respirable coal mine dust (Goodman and Organiscak 2002; Listak and Beck 2012; Reed et al. 2017). This roof bolting machine CAC research continues to the present day.

National Institute for Occupational Safety and Health (NIOSH) conducted a study which indicated that coal mine respirable dust overexposures are a concern for shuttle car operators when blowing face ventilation is used to ventilate the continuous miner face while cutting and loading coal. Table 1 summarizes the averages of the coal mine respirable dust exposure of shuttle car operators measured during continuous miner operation—cutting and loading coal—at different mining operations (Potts et al. 2011). In Table 1, straight cuts are defined as the continuous miner cutting straight into the entry. Right and left cuts are defined as the continuous miner cutting or turning a crosscut in the respective direction off the entry.

**Table 1:** Average coal mine respirable dust concentrations with 85% confidence intervals, measured at the location of shuttle car operators when the continuous miner cuts and loads cars (Potts et al. 2011)

Mine	Cut depth (m)	Straight cut (mg/m^3^)		85% CI	Right cut (mg/m^3^)		85% CI	Left cut (mg/m^3^)		85% CI
A	6.1	4.13	±	0.70	5.31	±	1.01	NA		NA
	12.2	6.39	±	1.50	3.55	±	0.58	NA		NA
D	6.1	0.75	±	0.36	NA		NA	NA		NA
	9.2	2.73	±	0.68	NA		NA	NA		NA
E	6.1	1.27	±	0.18	1.24	±	0.25	1.50	±	0.35
	12.2	1.15	±	0.23	1.13	±	0.29	1.10	±	0.45
F	6.1	1.77	±	0.24	NA		NA	NA		NA
	9.2	2.13	±	0.25	NA		NA	NA		NA

*CI* confidence interval, *NA* not available

These exposures occur while the shuttle car operator is operating downwind of the continuous miner, waiting to be loaded with coal. It can be seen, from Table 1, that many of the exposures exceed 1.5 mg/m^3^. While these exposures only occur during continuous miner cutting and loading cycles when the shuttle car is downwind of the miner, it can be seen that they may be high enough to result in overexposures.

Research on the CAC is being expanded to include a CAC for shuttle car operators to provide respiratory protection from respirable coal mine dust. Ambient mine air is filtered and blown over the operator through a plenum built into the shuttle car canopy. A new version of the CAC, specifically designed for the shuttle car, has been developed under a NIOSH contract by Marshall University and J.H. Fletcher [contract #200-2015-63485], and is based upon NIOSH design recommendations. NIOSH completed the required laboratory testing of the shuttle car CAC to determine its ability to reduce the shuttle car operators’ respirable coal dust exposure. This paper details results of testing the shuttle car CAC in 0.61 m/s (120 fpm), 2.0 m/s (400 fpm), and 4.3 m/s (850 fpm) ventilation airflows. Since results with 4.3 m/s (850 fpm) ventilation airflows were not satisfactory additional tests with modifications to the location and design of the CAC were conducted. Modifications included moving the CAC 22.86 cm (9 in.) forward of operator location and adding a 5.08 cm (2 in.) spoiler. These modifications provided satisfactory results, thus showing that a shuttle car CAC can be a viable dust control device for the protection of shuttle car operators to coal mine respirable dust.

### Testing

**Fig. 1:**
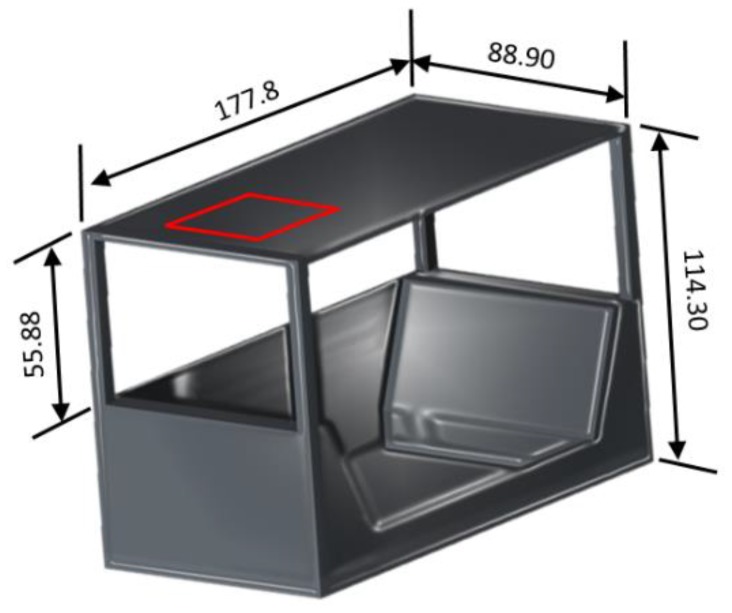
The center-drive shuttle car cab for the Joy 10SC32AA (dimensions in centimeters). The red outline shows the location of the canopy air curtain underneath the cab roof during testing (drawing by NIOSH)

**Fig. 2:**
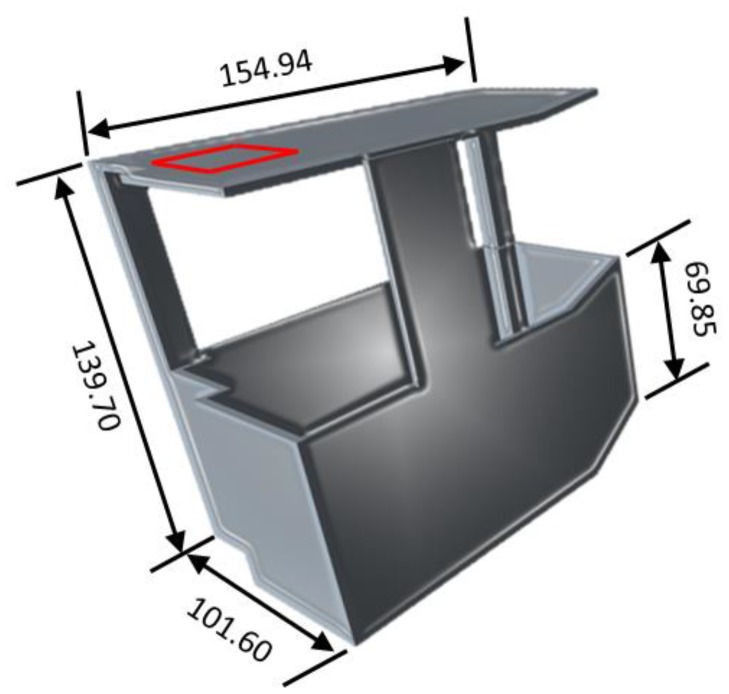
The end-drive shuttle car cab for the Joy 10SC32AB (dimensions in centimeters). The red outline shows the location of the canopy air curtain underneath the cab roof during testing (drawing by NIOSH)

The testing of the shuttle car CAC was conducted on a simulated shuttle car cab in an airflow corridor at NIOSH Pittsburgh Mining Research Division (PMRD) to determine effectiveness for dust reduction. The corridor dimensions were 2.29 m (90 in.) high by 1.98 m (78 in.) wide by an 18.9 m (62 ft.) long corridor. Two different shuttle car cab designs were evaluated—a center drive cab similar to that on a Joy 10SC32AA model shuttle car and an end-drive cab similar to that on a Joy 10SC32AB shuttle car. These two shuttle car models were found to be the most commonly used in underground coal mines. These designs are shown in Figs. 1 and 2. The dimensions are approximated from actual measurements of typical shuttle cars at an operating underground coal mine site. During setup, the cabs were placed in the center of NIOSH’s longwall gallery return airway at 25.4–30.5 cm (10–12 in.) above the floor as specified by the ground clearance for each shuttle car model. No obstructions to cab openings, such as wheel fenders, caging of cab openings, etc. were simulated.

**Fig. 3:**
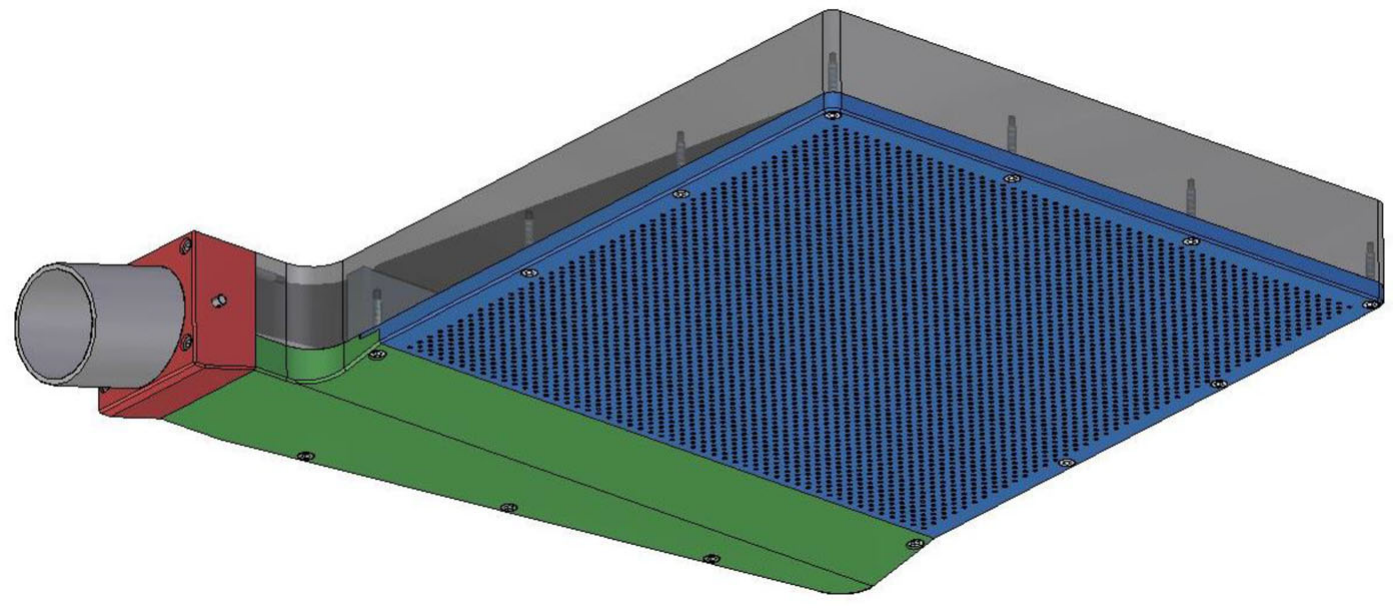
The canopy air curtain underneath the roof of the shuttle car (drawing by Marshall University and J.H. Fletcher, Inc.)

**Fig. 4:**
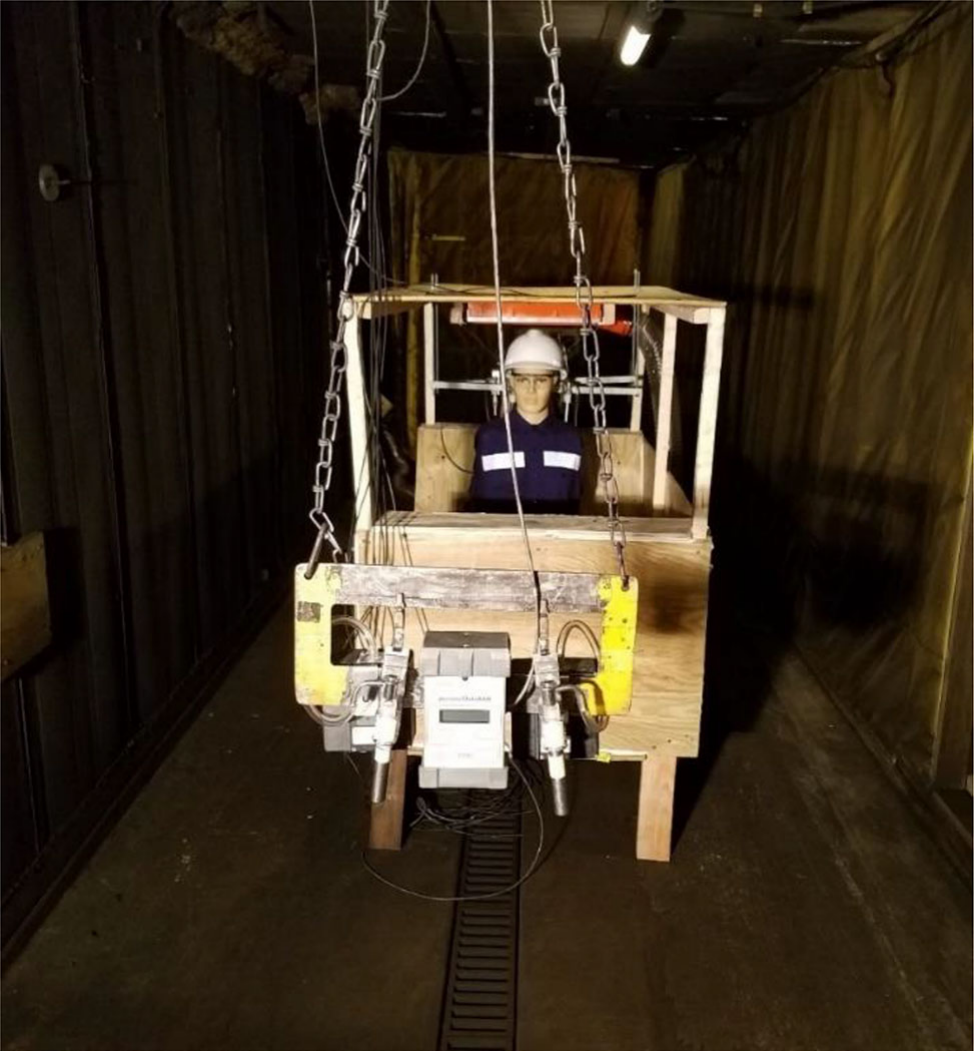
Photo showing the setup of the center-drive cab for testing with the manikin used for centering the CAC over the shuttle car operator

The CAC (Fig. 3) was attached onto the bottom side of the shuttle car roof (canopy) and centered over the seating area of the operator. For purposes of lab testing, all necessary components for generating airflow, including the blower, drive, and intake filter were set up outside the test area. Figure 4 shows the center-drive cab setup for testing.

### Sampling method

Both gravimetric and instantaneous samplers were used for testing the CAC for respirable dust control. Each gravimetric sampler consisted of an Escort ELF^®^ pump, a 10-mm Dorr-Oliver cyclone, and a 37-mm, 5-µm PVC filter. The Escort ELF pumps were calibrated to maintain 2.0 L/min airflow. The Thermo Scientific pDR-1000^®^was used as the instantaneous sampler. The typical sampling package was comprised of two gravimetric samplers and one instantaneous sampler. One sampling package was placed approximately 0.91 m (3 ft.) upstream of the shuttle car cab and another was placed 0.91 m (3 ft.) downstream of the shuttle car cab. These sampling packages monitored the respirable upstream and downstream dust concentrations to ensure consistent dust concentrations throughout the test.

**Fig. 5:**
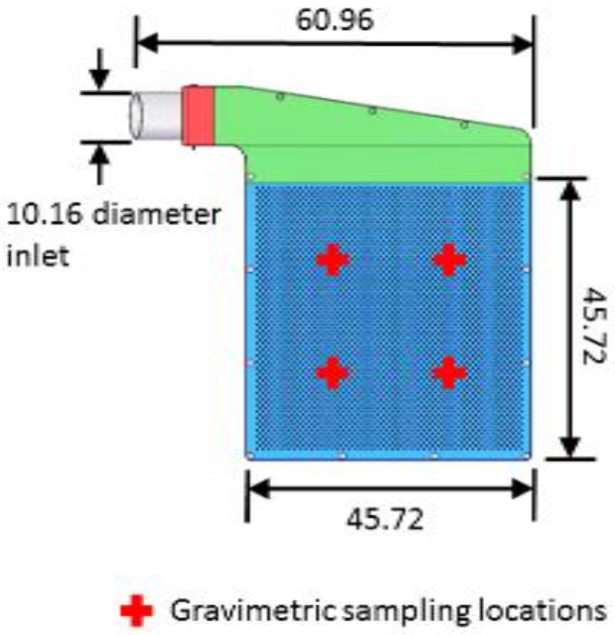
The canopy air curtain showing gravimetric filter locations (measurements on figure are in centimeters)

**Fig. 6:**
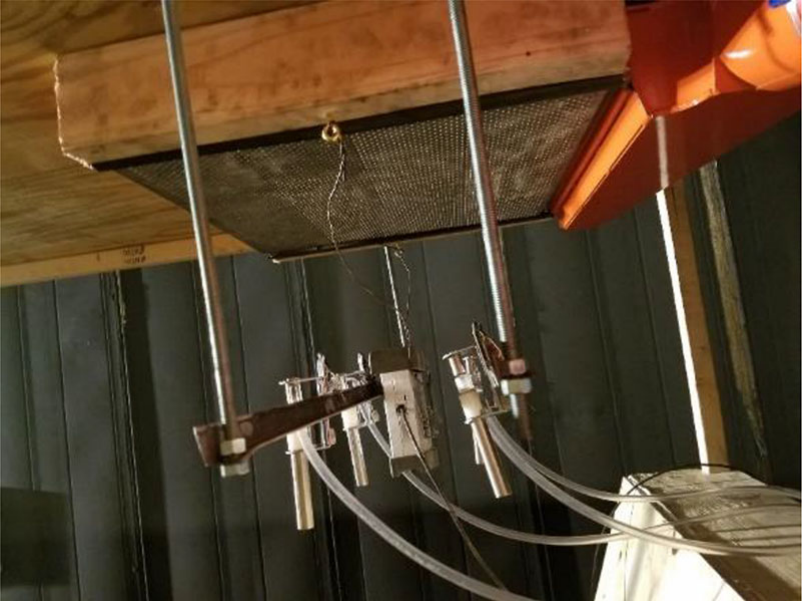
Photo showing the sampling locations, both the pDR-1000 and 4 gravimetric samplers, underneath the CAC

To test the CAC for dust control effectiveness, the sampling heads of four gravimetric samplers were placed at different locations at approximately 25.4 cm (10 in.) underneath the CAC (Fig. 5). A pDR-1000 was also placed in the center of the four gravimetric samplers underneath the CAC (Fig. 6). Later on during testing of the modifications to the CAC, continuous personal dust monitors (CPDM) Thermo Fisher Scientific Model 3600 were used in place of the gravimetric samplers due ease of obtaining immediate results.

The amount of air supply to the canopy was measured using a hot wire anemometer. Blower velocity measurements were conducted by inserting the anemometer at a port in a 1.52-m (5-ft) length of straight 10.2-cm (4-in.) PVC pipe connected to the blower outlet. The port was located approximately 1 m (40 in.) from the blower outlet to minimize turbulent airflow effects from the blower outlet.

### Test procedure

Three ventilation air velocities of 4.3 m/s (850 fpm), 2.0 m/s (400 fpm), and 0.61 m/s (120 fpm) were tested in the return section where the CAC test stand was located. The 0.61 m/s (120 fpm) represented the air velocity encountered when being loaded by the continuous miner, and the 4.3 m/s (850 fpm) represented the max air velocity encountered when tramming to the feeder. The 2.0 m/s (400 fpm) ventilation air velocity was tested as an approximate midway point between the other two ventilation velocities. A dust feeder was used to obtain an upwind respirable dust concentration targeting approximately 6.0 mg/m^3^. This concentration was selected because previous NIOSH studies have shown that this level of dust is encountered in the return of continuous miners using scrubbers (Colinet et al. 2013). The instantaneous sampler was used to monitor dust concentrations during testing.

Once the 6.0 mg/m^3^ concentration was achieved, the CAC blower was turned on to supply airflow to the plenum underneath the cab roof. Sampling was then started and tests were conducted for 30 min. Initially, three trials for each test were conducted, resulting in a total of nine tests for each cab type—three tests at 0.61 m/s (120 fpm), three tests at 2.0 m/s (400 fpm), and three tests at 4.3 m/s (850 fpm). The data was analyzed using the estimation of the mean of a population using a single sample (Natrella 1963) to determine the need for additional trials, which were added as needed.

The effectiveness of the CAC was determined by comparing the respirable dust concentrations from the four gravimetric filters underneath the CAC with the respirable dust concentrations from the two gravimetric filters upstream from the test stand. The following equation was used for calculating the respirable dust control:

1 $$\% \,reduction = \left[ {1 - \left( {\frac{Average\, canopy\,gravs}{Average\,upstream\,gravs}} \right)} \right] \times 100\%$$

where *% reduction* = respirable dust reduction in percentage. *Average canopy gravs* = average of the dust concentrations (mg/m^3^) from the four gravimetric filters underneath the canopy. *Average upstream gravs* = average of the dust concentrations (mg/m^3^) from the two gravimetric filters upstream of the test canopy.

During all testing, the blower velocity measurements taken in the 10.16 cm (4 in.) PVC pipe varied from 17.45 to 25.53 m/s (3436–5025 fpm). These measurements were only taken before and after the trial was completed. The average of the before and after measurements was used to calculate the velocity during the trial. Converting the velocities to air quantities showed that the blower provided anywhere from 0.14 to 0.21 m^3^/s (299–439 cfm) to the CAC. Correlation coefficients were calculated comparing the airflow quantities to the dust reductions. The results of the correlation calculations, − 0.38 for end drive cars and -0.53 for center drive cars, never showed a strong correlation (correlation coefficients > |0.75|) of airflow quantity provided by the blower to dust reductions provided by the plenum. The dry temperature ranged from 16.7 to 23.9 °C (62–75 °F), with the relative humidity ranging from 23.4 to 67.4%. The barometric pressure ranged from 991 to 1029 mbar.

### Results

In each case, for both center-drive and end-drive cabs, the column labeled “Count” represents the number of trials tested. Initially, three trials were tested. Additional trials were added based upon the equation (Natrella 1963):

2 $$n = \frac{{\sigma^{2} z_{\alpha = 0.05}^{2} }}{{d^{2} }}$$

where *n* = sample size required [number of trials required], *σ* = standard deviation of number of trials, *z*_*α*=*0.05*_ = standard normal distribution value = 1.64 at 90%^1^ confidence for two-tailed test, *d* = allowable error.

In reviewing all trials, the number of trials performed was sufficient with the allowable error for the resulting dust reductions set at ± 5%.

The dust control results from the testing are shown in Tables 2 and 3 along with associated statistics, such as standard deviation and upper and lower 95% confidence intervals. It can be seen that the canopy air curtain on the center-drive cab has a dust control efficiency of approximately 74% in 0.61 m/s (120 fpm) ventilation airflow. In 4.3 m/s (850 fpm) ventilation airflow, the dust reduction was very low at 16%, demonstrating that the dust control efficiency in high airflow velocities was reduced.

**Table 2:** Dust control reduction and associated statistics for center-drive shuttle car cabs at different ventilation airflows

Description	Vent airflow (m/s)	Average reduction (%)	Standard deviation	Count	Upper CI (95%)	Lower CI (95%)
Center drive	0.61	73.7	3.1	5	76.4	71.0
Center drive	2.03	42.6	2.7	3	45.6	39.6
Center drive	4.32	16.3	1.5	5	17.6	15.0

*CI* confidence interval

**Table 3:** Dust control reduction and associated statistics for end-drive shuttle car cabs at different ventilation airflows

Description	Vent airflow (m/s)	Average reduction (%)	Standard deviation	Count	Upper CI (95%)	Lower CI (95%)
End drive	0.61	82.8	1.4	5	84.0	81.5
End drive	2.03	38.6	6.5	5	44.3	32.8
End drive	4.32	6.2	3.4	6	8.9	3.5

*CI* confidence interval

For the end-drive cab, it can be seen that the canopy air curtain has a dust control efficiency of up to approximately 83% in 0.61 m/g (120 fpm) ventilation airflow. In 4.3 m/s (850 fpm) ventilation airflow, the dust reduction was reduced to 6%, demonstrating that its dust control efficiency in high airflow velocities will also be reduced for end-drive cabs.

Past research has shown that as the ventilation velocity increases, the dust control efficiency of the CAC decreases (Engel et al. 1987). The results of this testing proved no different. However, the contract requires the ability to reduce the shuttle car operator’s respirable coal dust exposure by at least 60% and poor performance during tramming—where relative velocities are highest—could result in efficiencies below this level.

### Improvement of canopy air curtain dust control efficiencies

Because the contract focuses on shuttle cars with center-drive cabs, subsequent testing to improve the dust control efficiency was completed on the canopy air curtain installed on center-drive cabs. During this testing personal dust monitors (PDM) were used. The PDM allows downloading of the dust concentration data, which provides for quicker turnaround of results compared to weighing gravimetric filters. Therefore, PDMs were used in place of all gravimetric samplers, while the pDRs were still used to provide instantaneous dust concentration readings for monitoring dust inside the test facility.

**Fig. 7:**
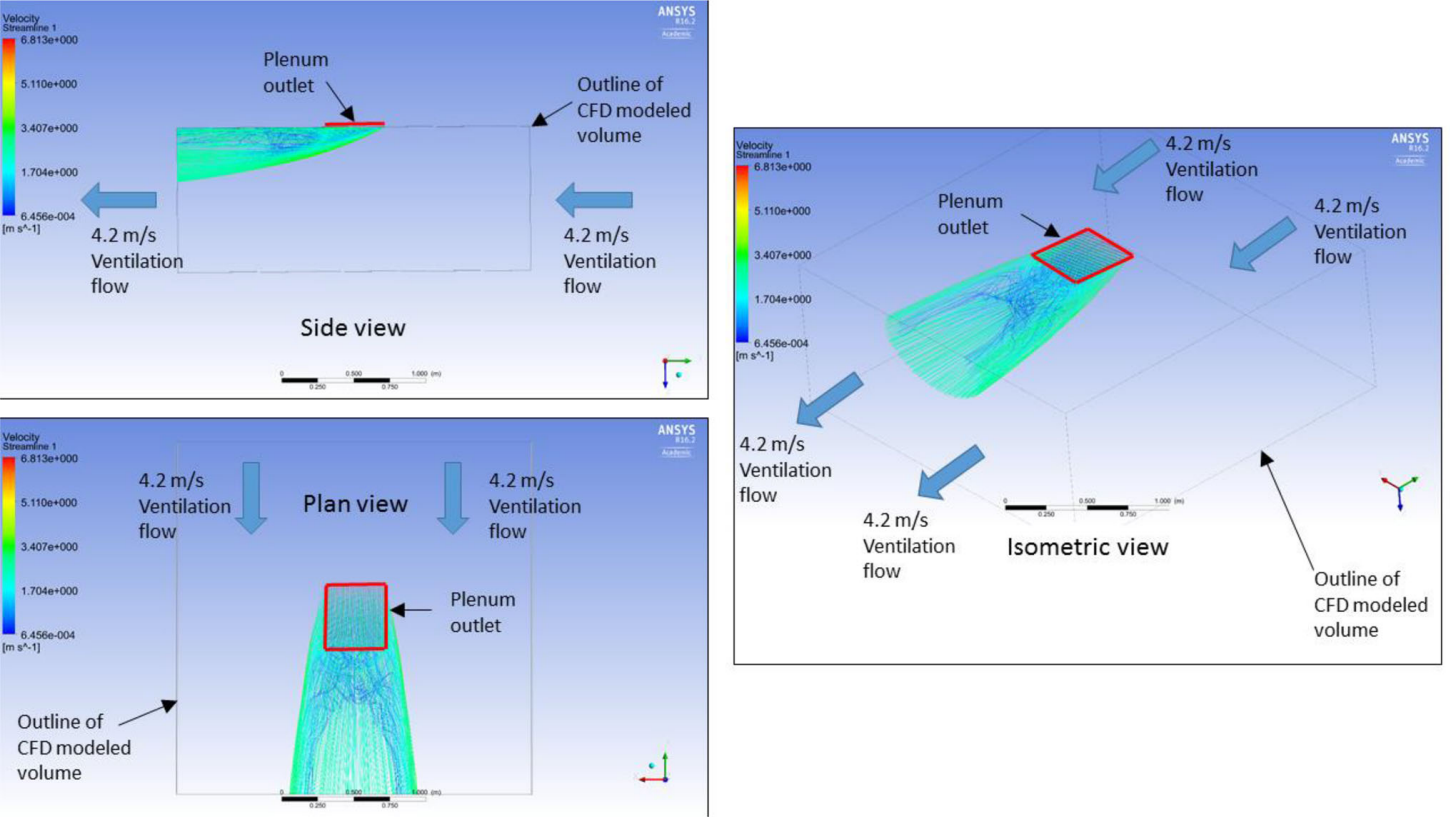
CFD simulation results showing airflow streams from shuttle car CAC plenum in 4.2 m/s ventilation airflow (Marshall University)

Reviewing the previous reports on canopy air curtain development from Marshall University/J.H. Fletcher & Co., Inc., the cross-sections resulting from computational fluid dynamic (CFD) simulations were studied for potential improvements. Of interest were the CFD model results of the canopy air curtain with approximately 4.2 m/s (830 fpm) lateral ventilation flow (Salem et al. 2016). Figure 7 presents the results of the CFD analysis showing results in side, plan, and isometric views. The CAC plenum is centrally located atop the CFD modeled volume and is outlined in red. The airstream lines (blue to light green color) are shown emanating from the plenum with the streamflow immediately pushed downstream due to the high 4.2 m/s (830 cfm) ventilation flow. Normally, airstream flow from the plenum in low velocity ventilation air emanates straight from the plenum with no deflection. The alignment of these airstream lines due to the deflection caused by the high 4.2 m/s (830 cfm) ventilation airflow demonstrates that the airflow protection the canopy offers to the worker seems to shift downwind in high-velocity ventilation airflows. From the observation of the different views displayed in this figure, generated by CFD, the canopy air curtain was shifted approximately 22.86 cm (9 in.) forward of the shuttle car operator’s position, and a front spoiler was added in an attempt to redirect ventilation airflow to improve its performance for dust control.

**Fig. 8:**
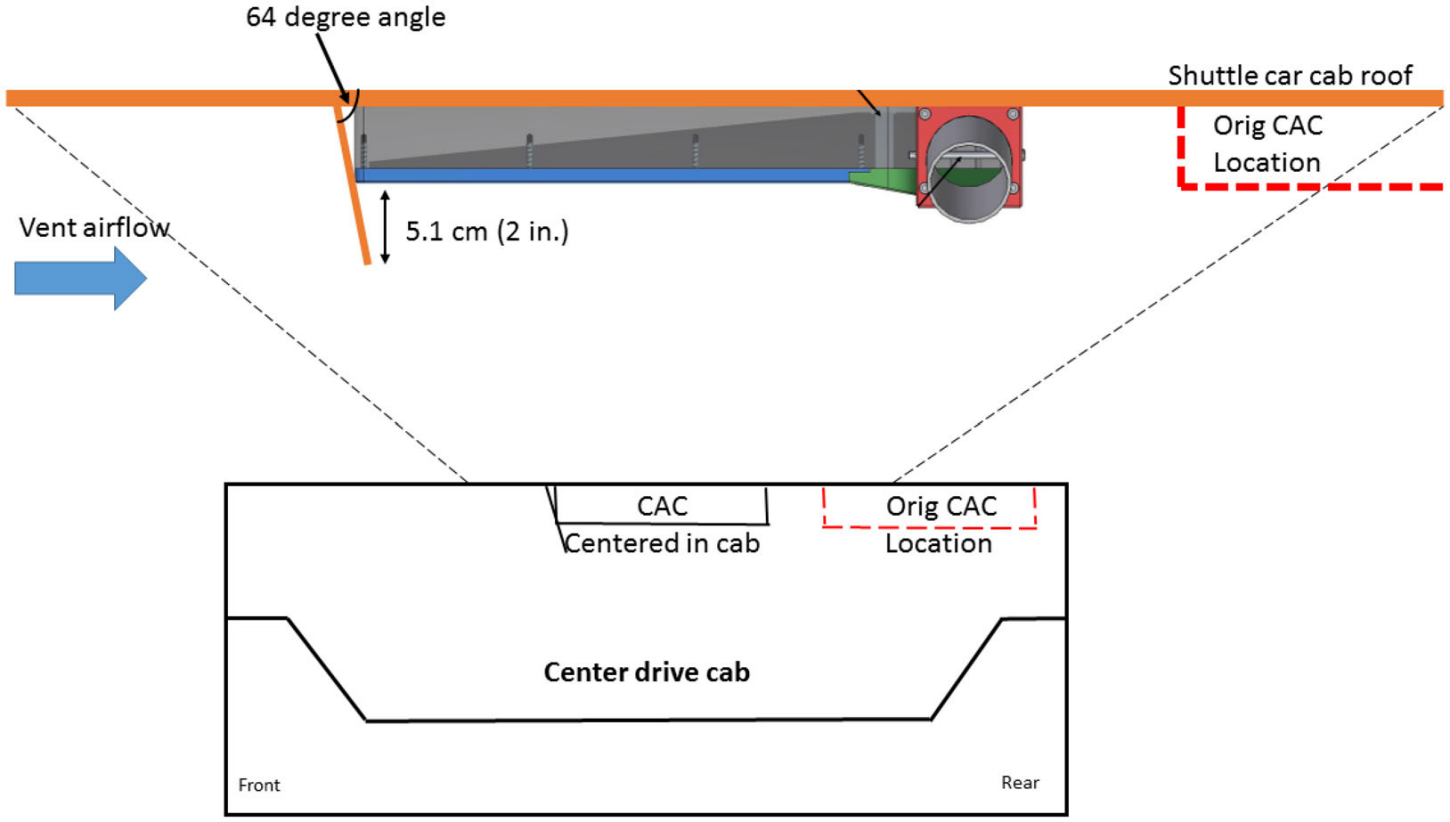
Canopy air curtain underneath cab roof displaying spoiler to redirect ventilation airflow

The front spoiler, consisting of a 12.7–15.2 cm (5–6 in.) wide piece of plywood that extended across the entire width of the center-drive cab, was added to the roof of the cab. Additionally, it was oriented at 64^o^ from the horizontal and extended approximately 5.08 cm (2 in.) below the canopy plenum outlets as shown in Fig. 8. The first series of tests placed the canopy centrally in the cab underneath the cab roof with a front spoiler. The sampling locations were not moved with the CAC and were located over the operator position. However, for this first series of tests, two additional samplers were added in front of the existing sampling rows to include sample locations 25.4 cm (10 in.) directly underneath the CAC (Fig. 10).

**Fig. 9:**
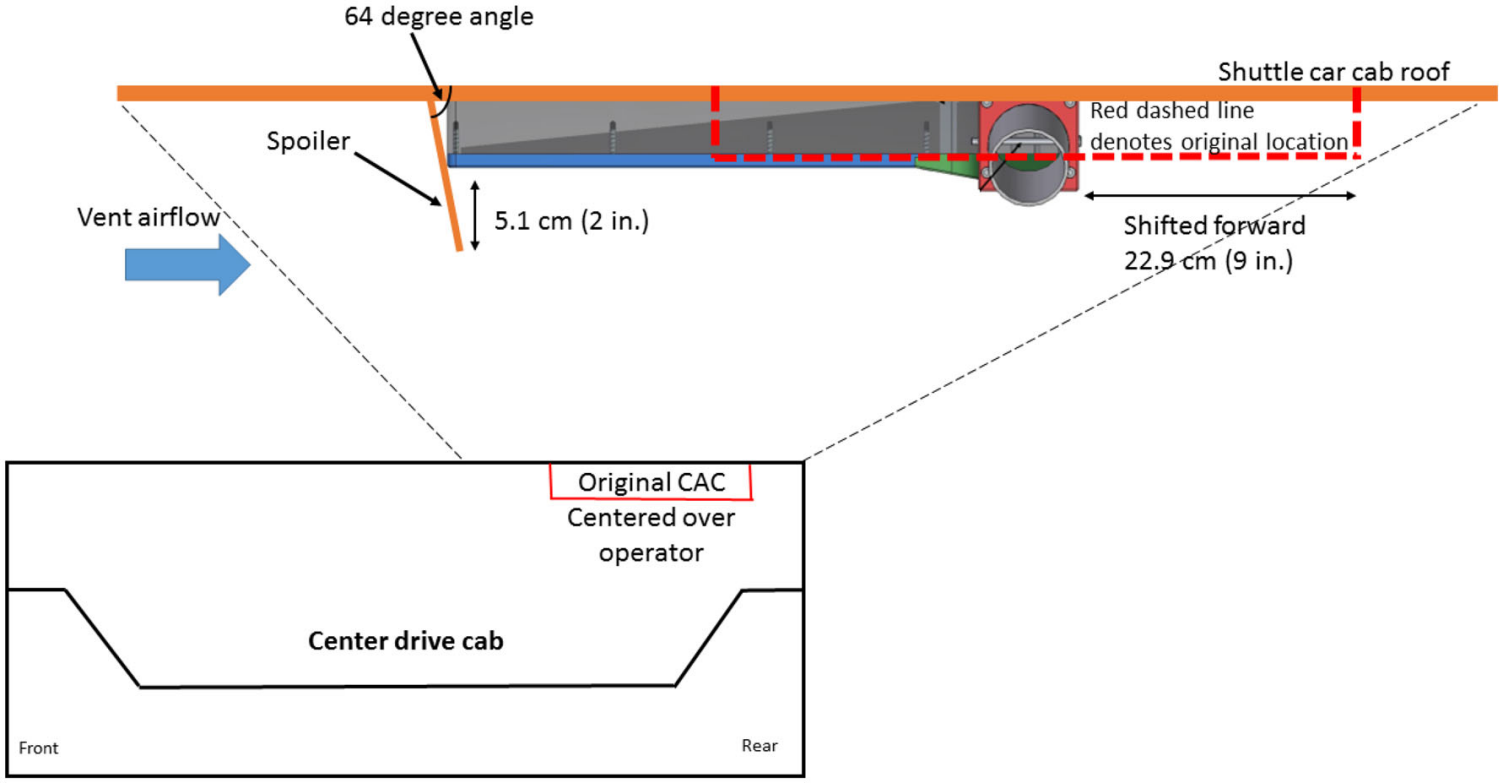
Canopy air curtain underneath cab roof, displaying forward shift of canopy location 22.9 cm (9 in.) and spoiler to redirect ventilation airflow

**Fig. 10:**
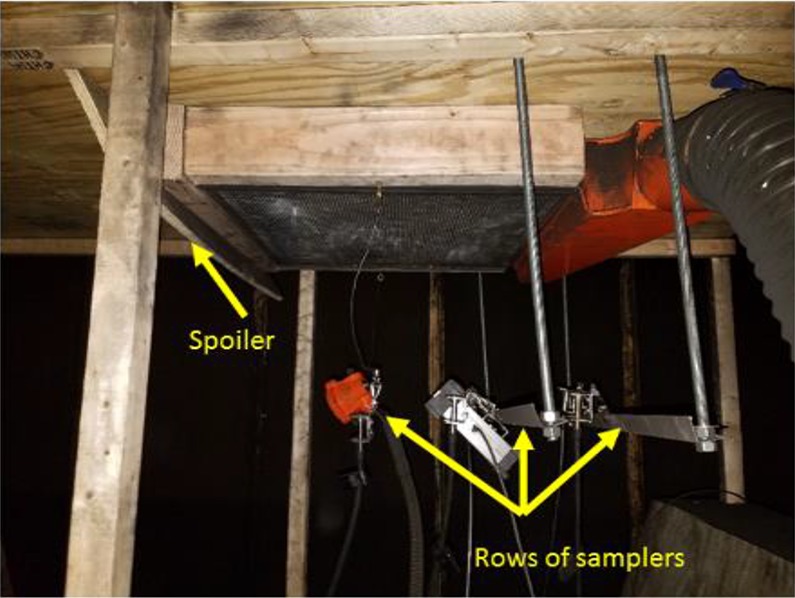
Photo showing additional sampling row added to provide full sampling coverage underneath the CAC. Spoiler is shown on the front of the CAC

The second series of tests used the same spoiler, but moved the canopy air curtain from the centered position to a location 22.9 cm (9 in.) forward from the original over-operator position (Fig. 9). Again, the sampling locations were not moved with the CAC. They were left in place, centered over the operator position. However, for this second series of tests, one additional row of samplers was added to include sample locations 25.4 cm (10 in.) underneath the CAC (Fig. 10). During testing two CPDM per row were used, totaling six CPDMs, replacing the gravimetric samplers. In all subsequent analysis for CAC respirable dust reductions, *Average Canopy Gravs* from Eq. (1) averaged the results of all six samplers underneath the CAC area of influence. Results of these tests can be seen in Table 4.

**Table 4:** Dust control reduction and associated statistics for a center-drive shuttle car at different ventilation airflows with different canopy configurations as shown in Figs. 8 and 9

Description	Vent airflow (m/s)	Average reduction (%)	Standard deviation	Count	Upper CI (95%)	Lower CI (95%)
Canopy with spoiler, canopy centered underneath cab	4.32	36.2	0.43	3	36.7	35.7
Canopy with spoiler, canopy moved 22.9 cm (9″) forward of operator	0.61	70.4	2.09	6	72.1	68.7
Canopy with spoiler, canopy moved 22.9 cm (9″) forward of operator	2.03	65.9	0.42	6	66.3	65.6
Canopy with spoiler, canopy moved 22.9 cm (9″) forward of operator	4.32	51.3	6.78	3	59.0	43.6

*CI* confidence interval

The canopy centered underneath the cab increased the average dust reduction to 36% (from 16%). Reviewing the CFD analysis of the CAC showed that moving the CAC only 22.86 cm (9 in.) forward might provide better results. Therefore, tests that centered the CAC underneath the cab were discontinued and tests that moved the CAC 22.86 cm (9 in.) forward were tested. Test results showed that the combination of the spoiler and moving the CAC 22.86 cm (9 in.) forward of the operator seemed to allow the CAC to perform better in the high ventilation airflows, having a 51% average reduction in 4.3 m/s (850 fpm). The CAC also performed better with 66% dust reduction at 2.0 m/s (400 fpm) ventilation airflow. At 0.61 m/s (120 fpm), the respirable dust reduction was 70%. The 51% dust reduction at 4.3 m/s (850 fpm) is a substantial increase in dust reduction from the canopy without any modifications to the CAC itself. In reviewing all results, the number of samples was sufficient when setting the allowable error at ± 5% in dust reduction. It is assumed that these results will translate to equivalent results for the end-drive cabs.

**Fig. 11:**
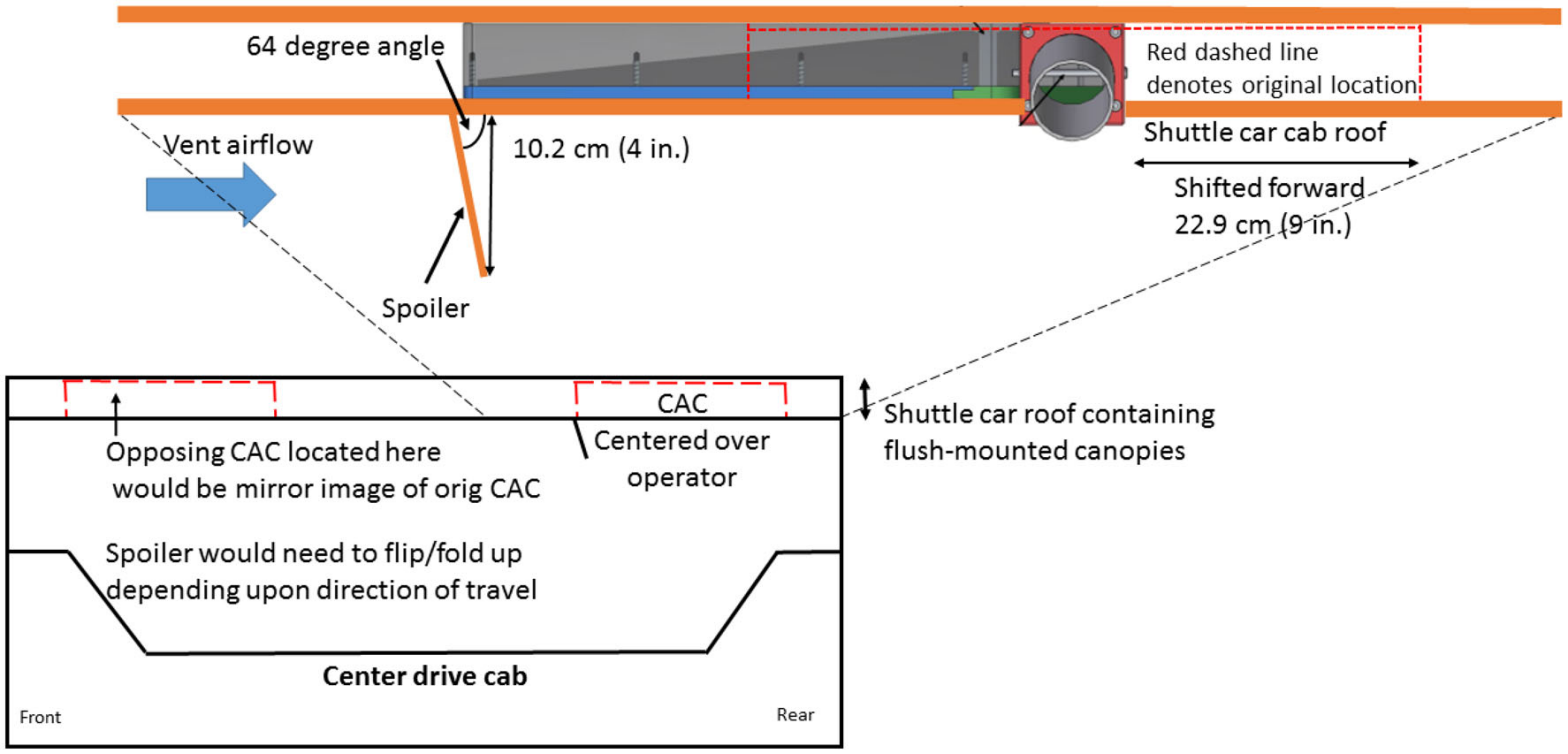
Canopy air curtain built into cab roof, displaying forward shift of canopy location (9 in. forward) and spoiler to redirect ventilation airflow

A single test with the CAC moved 22.86 cm (9 in.) forward with a spoiler and an additional dummy CAC installed in front, which represented the planned design of two CACs installed underneath the cab roof, showed that the dust reduction dropped to 35% at 4.3 m/s (850 fpm), similar to the results in Table 4 with the CAC centered underneath the cab roof. Additional testing, which simulated the two canopies flush mounted onto the roof with one spoiler with a 10.16 cm (4-in.) drop instead of a 5.08 cm (2 in.) drop (Fig. 11), showed better results [a 55% reduction at 4.3 m/s (850 fpm)].

The results in Table 5 show that the canopies flush mounted onto the shuttle car roof will have better success in reducing respirable dust for the shuttle car operator; similar results, as shown in Table 4, were provided by the CAC with the spoiler moved 22.86 (9 in.) forward.

**Table 5:** Dust control reduction and associated statistics for center-drive shuttle car for an improved canopy configuration shown in Fig. 11

Description	Vent airflow (m/s)	Average reduction (%)	Standard deviation	Count	Upper CI (95%)	Lower CI (95%)
Two canopies, 22.9 cm (9″) forward, mounted underneath roof with spoilers on both	4.32	26.6	NA	1	NA	NA
Two canopies, 22.9 cm (9″) forward, mounted underneath roof with spoiler on operator position	4.32	35.4	NA	1	NA	NA
Two canopies, 22.9 cm (9″) forward, flush-mounted underneath roof with spoiler on operator position	4.32	54.8	1.26	3	56.2	53.4

*CI* confidence interval

### Conclusions

Laboratory tests conducted with the canopy air curtain as currently designed, and with its planned location on the shuttle car cab, has shown to be more than sufficient for dust reductions in airflow velocities up to 0.61 m/s (120 fpm), with reductions of 74% (center drive) and 83% (end drive). However, at 4.3 m/s (850 fpm) ventilation airflow, the reductions were very low at 16% (center drive) and 6% (end drive) and not sufficient to meet the contract requirements of 60% dust reduction during the entire operation of the shuttle car.

To improve dust reductions from the CAC, modifications to the canopy locations were tested. Installing a spoiler and moving the CAC 22.86 cm (9 in.) forward from the operator location seem to provide the best improvement in the performance of the CAC. Dust reductions of 70% at 0.61 m/s (120 fpm), 66% at 2.0 m/s (400 fpm), and 51% at 4.2 m/s (850 fpm) ventilation airflows were achieved. Although the 60% dust reduction threshold was not met at 4.2 m/s (850 fpm), a 51% reduction is close and is sufficient to proceed with field testing. In addition, the 4.2 m/s (850 fpm) air ventilation quantity threshold was originally based upon a mine ventilation velocity measured in an intake entry and the maximum shuttle car speed of 9.6 km/hr (6 mph) (Joy Global 2016). Recent studies completed by NIOSH show that the maximum air ventilation quantity threshold may not be equal to mine intake air plus the maximum shuttle car speed (Shahan and Reed 2018), and relative velocity may be significantly lower than 850 fpm in mines. In addition, the higher airflow a shuttle car encounters is generally associated with lower dust concentrations. In fact, the shuttle car operators’ highest respirable dust exposure occurred when being loaded by the continuous miner in blowing face ventilation. Therefore, the modification of moving the CAC 22.9 cm (9 in.) forward and flush mounted onto the shuttle car cab roof should be sufficient to achieve the targeted 60% reduction.

These laboratory tests showed that the canopy air curtain for the shuttle car is successful at reducing respirable coal mine dust exposure at low ventilation velocities. Redesign of the CAC to shift it forward 22.9 cm (9 in.) with a spoiler and flush mount it onto the shuttle car roof should successfully protect the shuttle car operator from respirable coal mine dust as seen by the results from these tests. Other options to improve the dust control efficiency of the CAC could be evaluated, such as increasing the airflow to the CAC and/or enlarging the size of the CAC to have a single CAC over the entire canopy. But, these enhancements are probably not necessary for improving dust control efficiency. The laboratory testing on the shuttle car CAC demonstrates that it can successfully protect the shuttle car operator by reducing exposure to coal mine respirable dust. A field test is being planned to test the CAC at a mine site that uses blowing face ventilation.

## Footnotes

1 The confidence level was relaxed to 90% from the commonly used 95% level for this lab testing. While lab testing has much better control over influences on testing, there are still factors which cannot be controlled such as air properties, dust feed moisture content, airflow fluctuations, dust feed variations, etc. Additionally, laboratory availability can place time constraints limits on lab testing limiting the number of trials completed. A 90% confidence level is still a robust standard.
